# Mandibular Inflammatory Myofibroblastic Tumors: A Literature Review with a New Case Presentation

**DOI:** 10.3390/curroncol33070375

**Published:** 2026-06-23

**Authors:** Cristina Benites, Kirollos Armosh, Edgar D. Uribe Sanchez, Lauren A. Ruddocks, Peter T. Dziegielewski

**Affiliations:** 1 Department of Otolaryngology–Head and Neck Surgery, University of Florida, Gainesville, FL 32611, USA; cb3016@mynsu.nova.edu (C.B.); or euribesanchez@umc.edu (E.D.U.S.); 2 University of Florida College of Medicine, University of Florida, Gainesville, FL 32611, USA; armosh.k@ufl.edu; 3 Department of Pathology, Immunology and Laboratory Medicine, University of Florida, Gainesville, FL 32611, USA; 4 University of Florida Cancer Center, Gainesville, FL 32611, USA

**Keywords:** inflammatory myofibroblastic tumor, anaplastic lymphoma kinase, Jaw in a Day

## Abstract

**Aim:** Inflammatory myofibroblastic tumor (IMT) is a rare mesenchymal neoplasm with few reported mandibular cases. This review aims to describe the clinical characteristics, surgical management and outcomes of 13 mandibular IMT cases, and to introduce the Jaw in a Day (JIAD) approach as a novel surgical option. **Methods**: PubMed, Web of Science and Embase were searched systematically. From an initial pool of 261 articles, 13 studies met the inclusion criteria and were reviewed. **Results**: Thirteen mandibular IMT cases were identified in the literature. A new case is also presented: a 15-year-old female treated with surgical excision using the JIAD approach. At 12-month follow-up, oral function was restored with no disease recurrence. **Discussion**: Mandibular IMT is exceedingly rare. The JIAD approach offers immediate reconstruction with satisfactory functional outcomes, as supported by both the current case and the reviewed literature. **Conclusions**: The JIAD surgical approach may represent an effective management strategy for mandibular IMT. Further cases are needed to validate this approach and establish standardized treatment protocols.

## Simple Summary

This study reviews 13 previously reported cases of inflammatory myofibroblastic tumors (IMTs) involving the mandible, showing that this type of mandibular tumor, while uncommon, can be effectively treated with surgical resection, yielding favorable outcomes. This review also examines the imaging characteristics, histopathologic differential diagnosis, and molecular markers critical for distinguishing mandibular IMTs. In addition to this review paper, we present the case of a 15-year-old female with a mandibular IMT who underwent tumor removal and immediate reconstruction using the “Jaw in a Day” technique. The patient regained normal oral function and had no tumor recurrence after 12 months. This surgical approach allows for immediate jaw reconstruction and beneficial functional results.

## 1. Introduction

Inflammatory myofibroblastic tumors (IMTs) are a heterogeneous group of rare neoplasms composed of myofibroblastic and fibroblastic spindle cells that contain an inflammatory infiltrate of plasma cells, lymphocytes and/or eosinophils [1]. Patients typically present with a slowly growing, well-defined swelling over months to years. Although benign, IMTs can mimic malignancies clinically and radiographically. They have the potential for aggressive progression or vascular invasion. IMTs also have a tendency for local recurrence and present a small risk of distant metastasis [2].

IMTs primarily develop in the lungs, liver and abdominal regions. In the head and neck region, IMTs are rare but can affect the aero-digestive tract and major salivary glands. Extremely rare cases have been observed in the mandible [2,3].

Numerous etiologies have been implicated in the pathogenesis of IMTs, including prior infection, autoimmune diseases, foreign body reactions, and previous radiation therapy [4]. The heterogeneous nature of these tumors and their overlapping clinical features with both benign and malignant lesions make early and accurate diagnosis challenging. In the mandible specifically, IMTs on imaging may present as aggressive lytic bone lesions that can mimic a range of odontogenic and non-odontogenic pathologies, including periapical (radicular) cysts, ameloblastomas, central giant cell granulomas, fibrous dysplasia, Langerhans cell histiocytosis, osteosarcoma, and metastatic disease [5,6]. Clinicians encountering a large or aggressive-appearing lytic lesion of the mandible, particularly one that does not respond to conventional endodontic or antibiotic therapy, should maintain a high index of suspicion for IMT among other spindle cell neoplasms. A case of mandibular IMT that initially mimicked apical periodontitis has been described, underscoring the importance of considering this diagnosis when lesions behave atypically or fail to resolve with standard treatment [7]. Although the present review focuses on mandibular IMTs, intraosseous IMTs have also been reported in the maxilla. An intraosseous inflammatory pseudotumor of the maxilla presented as a destructive bony lesion, further illustrating the diagnostic challenge posed by these tumors in the craniofacial skeleton [8].

A total of 13 cases of mandibular IMT have been reported to date. The aim of this study is to review all published mandibular IMT cases and to present a new case, with a focus on surgical management using the JIAD technique and its associated outcomes. This study focuses on presenting a comprehensive review paper of published cases in the literature and a new case to further illustrate management and outcomes.

## 2. Materials and Methods

An authorization to use and disclose protected health information (PHI) for education, publication and public display consent form was obtained from the patient, and a review of the medical records of the patient was conducted. We designed a dual approach to test our hypothesis that mandibular inflammatory myofibroblastic tumors managed with complete surgical resection and immediate reconstruction result in restored oral function and a disease-free status at one year follow-up. 

**Figure 1:**
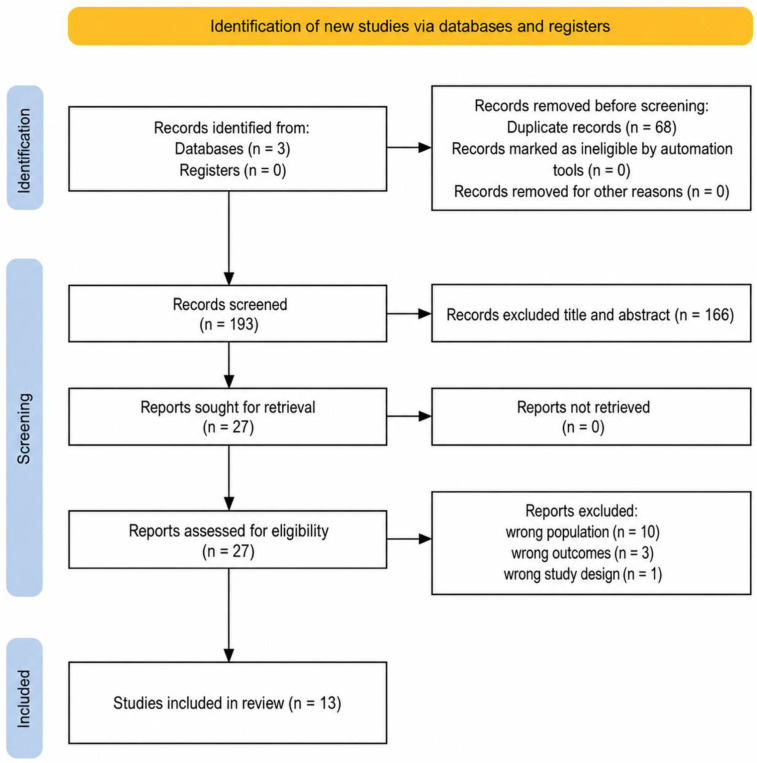
Review paper search strategy.

A paper review of the literature was conducted using PubMed, Web of Science and Embase. Search terms included were “Inflammatory Myofibroblastic Tumor” or “IMT” and “Head and Neck” or “Mandible”. Given the limited yield, no restrictions on publication year were applied to the search. Cases of IMT located in the head and neck region were reviewed, with a specific focus on those involving the mandible. Article references within identified reports with further potential cases were also gleaned. The initial search yielded 257 articles, and 4 additional articles were identified within the references, resulting in 261 articles in total. After removal of 68 duplicate records, 193 records underwent title and abstract screening. Of these, 166 records were excluded based on title and abstract review, leaving 27 reports sought for retrieval and assessed for eligibility. Following full text review, 10 reports were excluded for wrong populations, 3 for wrong outcomes, and 1 for wrong study design. Ultimately, 13 studies met the inclusion criteria. The search strategy is outlined in Figure 1.

## 3. Results

This study included a paper review that yielded 13 patients, along with a case presentation of one 15-year-old patient with an IMT diagnosis. The interventions in all cases were surgical resections, except in one case where the specific treatment was not reported. Outcomes were measured by the restoration of oral function and a disease-free state at 12-month follow-up. The findings of the case presentation presented in this study were compared to those of mandibular IMT cases found in the paper review. This study is designed as a combined review paper and case presentation. One case of IMT involving the mandible was identified at the University of Florida/Shands, and 13 more were identified in the literature. All 14 cases are summarized in Table 1.

### 3.1. Case Presentation

**Figure 2:**
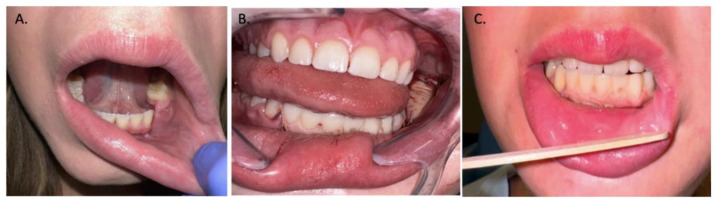
(**A**) Intraoral view of stable depression along anterior left inferior alveolar ridge; (**B**) intraoperative view; (**C**) one month post-operative view.

**Figure 3:**
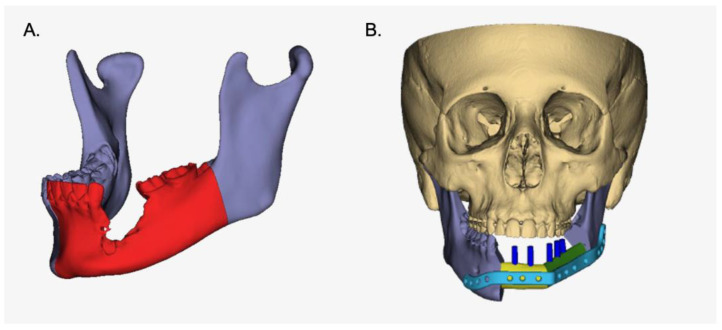
(**A**) 3D virtual surgical planning reconstruction with involved mandibular resection segment (red); (**B**) virtual surgical planning of mandibular reconstruction plan of left fibula graft (yellow and green) with dental implant cylinders (blue) and patient-specific plate for mandible (light blue).

**Figure 4:**
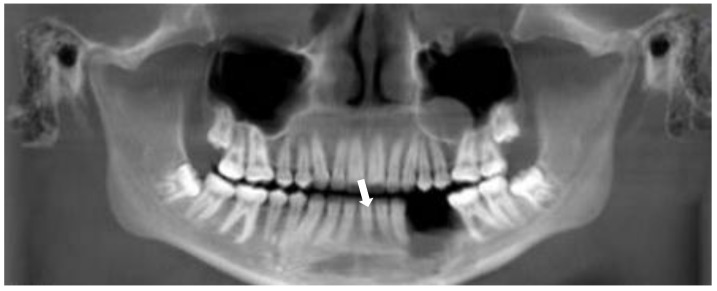
Pre-operative radiograph demonstrating ill-defined radiolucent lesion in left anterior-to-premolar mandibular body. White arrow indicates lesion.

**Figure 5:**
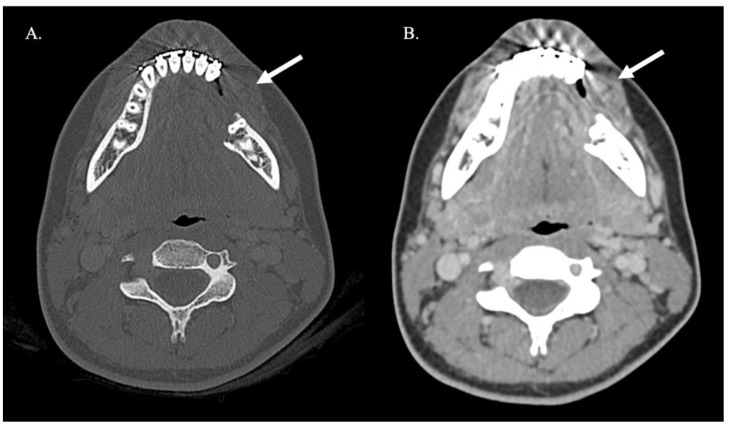

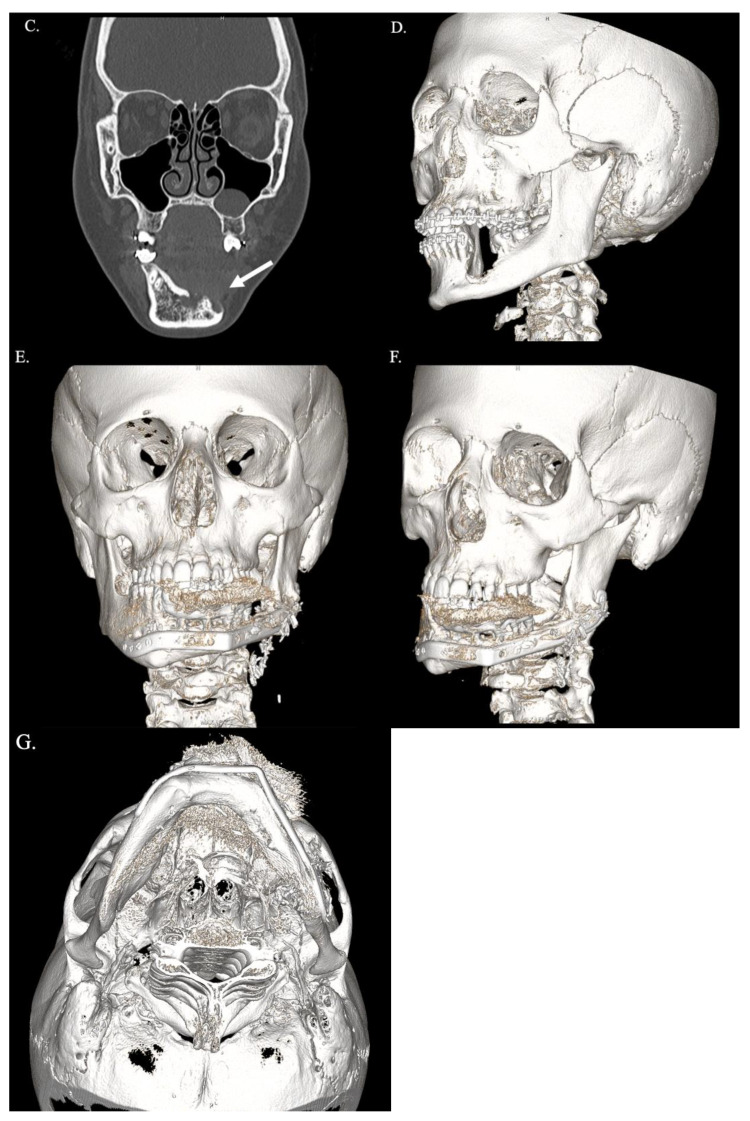
Pre-operative contrast-enhanced CT imaging of mandibular lesion. Axial CT with contrast, bone reconstruction/window (**A**). Axial CT with contrast, soft tissue reconstruction/window (**B**). Coronal CT with contrast, bone reconstruction/window (**C**). Three-dimensional multidetector computed tomography (MDCT) reconstruction (oblique lateral view) demonstrating osseous anatomy of mandible and involved mandibular segment (**D**). 3D CT scans at 2 years post-surgery (**E**–**G**). Coronal view demonstrating reconstruction place positioned as planned, dental implants and prosthesis in place (**E**). Three-quarter coronal view showing excellent neo-mandibular contour. (**F**) Axial view showing complete ossification at osteotomy sites (**G**). Contrast-enhanced CT does not demonstrate suspicious soft-tissue focal enhancement at periphery of mandibular tumor. White arrows indicate tumor on images (**A**–**C**).

**Figure 6:**
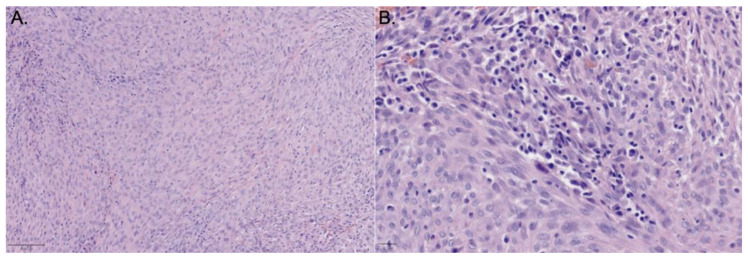
Histologic images from diagnostic biopsy specimen. (**A**) Hypercellular tumor composed of fascicles of spindle-shaped cells in collagenous stroma, with interspersed inflammatory cells (H&E, ×10); (**B**) inflammatory infiltrate composed primarily of lymphocytes and plasma cells (H&E, ×40).

A 15-year-old female presented with progressive pain and paresthesia in the left premolar mandible for 9 months. A depression had formed along the alveolar ridge (Figure 2A); hypesthesia was present around this area and the chin. The lesion was seen on panoramic radiograph (panorex) and computed tomography (CT). An incisional biopsy returned an inflammatory myofibroblastic tumor harboring an ALK rearrangement, confirmed by an outside institution with cytogenetic reference testing. The tumor was deemed resectable with negative margins, and the team proceeded with surgical excision rather than neoadjuvant crizotinib. Virtual surgical planning was performed (Figure 3 and Figure 4). Contrast-enhanced CT demonstrated an infiltrative, minimally enhancing, low-attenuation lesion within the marrow space of the left hemimandible, consistent with a predominantly solid fibroblastic process and a pattern of intermediate biologic activity. The lesion occupied the medullary space, measuring approximately 16.9 × 17.4 × 12.4 mm (anteroposterior × mediolateral × craniocaudal), extending from just across the midline in the mental symphysis along the lingual plate of the mandible as far to the right as the position of the canine tooth socket, and on the left side to the marrow space between the first and second molars. The disease was chronically erosive and infiltrative. The appearance of the medial and lateral cortical plates suggested that the disease may have been contained by the periosteum of the mandible without definitive invasion of the floor of the mouth (Figure 5A–D). A separate non-contrast CT was not available, MRI evaluation was limited by orthodontic hardware artifact, and delayed enhancement was not reported on the available CT protocol. The mandibular canal/inferior alveolar nerve relationship and a discrete vascular component were not specifically described in the radiology report. Histological examination revealed a hypercellular tumor composed of fascicles of spindle-shaped cells embedded in a collagenous stroma, with interspersed inflammatory cells primarily consisting of lymphocytes and plasma cells intermixed with giant cells (Figure 6A,B). The lesion displayed more than one histologic pattern, predominantly a hypercellular compact spindle cell pattern, with scattered hypocellular fibrous, myxoid and vascular areas. Mitotic activity was 5 mitoses per 2 mm^2^. Immunostains showed that the lesional cells were positive for both anaplastic lymphoma kinase (ALK) (5A4) in 74% of nuclei and smooth muscle actin (SMA). Immunostains were negative for AE1/AE3, desmin, myogenin and CD34. The resection specimen showed clear margins, with no perineural or vascular invasion. These findings were consistent with IMT.

This patient underwent a composite resection and fibula free flap reconstruction. Virtual surgical planning with immediate dental implantation and a dental prosthetic was performed (Figure 3). This “Jaw in a Day” (JIAD) procedure provided the patient with full oral reconstruction and function at the time of her curative surgery [4,9].

**Figure 7:**
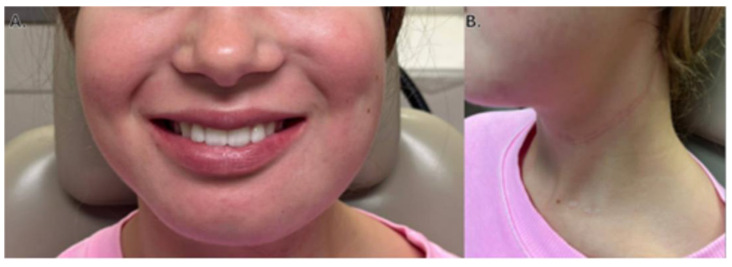
(**A**) 12-month follow-up view; (**B**) neck incision at 12 months post-op.

The patient remained on tube feeds for 6 days and then commenced a full soft oral diet. The patient was discharged on day 7 without complications. At the 12-month follow-up, she had undergone dental prosthetic adjustments and was enjoying a full oral diet. She is disease-free (Figure 7A,B).

### 3.2. Literature Review Results

This paper review demonstrates 13 cases of IMTs with mandibular presentation (Table 1). The mean age was 37 years (range: 11–82), and the majority of patients were female (8/13, 62%). Tumor sizes were reported in 10 cases, with dimensions ranging from 1.8 × 1.1 cm to 5 × 5 cm. The posterior mandible (retromandibular region) was the most common location, accounting for 69% of cases (9/13), while the remaining 31% (4/13) involved the alveolar region. Among the 11 cases with available follow-up data (range: 1–10 years), no recurrence was identified in any case. Tumor resection was performed with clear margins in most cases, with no indications of metastases or aggressive local behavior at follow-up.

**Table 1:** Summary of cases of mandibular IMT.

Author(s), Year	Study Design	Sample Size	Age	Sex	Initial Presentation	Size	Recurrence	Follow-Up Time	Treatment	Radiological Imaging Findings
Zegarelli et al., 1974 [10]	Case Report	1	56	F	Retromandibular	2.1 × 1.0 × 0.5 cm	No	1 year	Surgical excision with rib graft reconstruction	Standard X-ray: large radiolucent lesion (4.0 × 5.0 cm) described as ‘dental granuloma’ with destruction of alveolar ridge; CT/MRI/CBCT: not performed (predates CT era)
Earl et al., 1993 [11]	Case Report	1	44	M	Retromandibular	N/A	No	2 years	Radical curettage and extraction of 2nd and 3rd molars	Standard X-ray: vertical impaction of third molar with visible proliferative soft tissue; CT: soft tissue swelling involving mandible extending from angle to midline of pterygoid plates with no bony involvement; MRI: not reported
Inui et al., 1993 [12]	Case Report	1	63	M	Retromandibular	4 × 5 cm	No	2.5 years	Tumor enucleation	Standard X-ray: no odontogenic source or salivary calculus identified; CT: oval, circumscribed mass with central necrotic tissue in anterior part of submandibular gland; MRI: circumscribed mass suggesting origin from submandibular gland, no lymphadenopathy
Ide et al., 1998 [13]	Case Report	1	43	F	Retromandibular	2.3 × 1 cm	No	1 year	Initial curettage followed by re-excision of biopsy site and surrounding tissue	Standard X-ray: no evidence of bone erosion; CT/MRI: not reported
Fang et al., 2004 [14]	Case Report	1	23	M	Retromandibular	5 × 4 cm	No	2 years	Left commando procedure with modified supraomohyoid radical neck dissection and pre-operative tracheostomy	Standard X-ray (panoramic): radiolucent, osteolytic lesion of left posterior mandible with irregular border; CT (w/IV contrast): osteolytic lesion 5 × 4 cm with soft tissue mass extending into floor of mouth, mandibular canal intact, no enlarged lymph nodes; MRI: not reported
Brooks et al., 2005 [15]	Case Report	1	82	F	Alveolar	5 × 5 cm	No	18 months	Total excision	Standard X-ray (panoramic): soft tissue image overlying resorptive defect along superior aspect of left body of mandible; follow-up panograph at 18 months showed osseous regeneration at previous site of resorption; CT/MRI: not reported
Oh et al., 2008 [16]	Case Report	1	20	F	Retromandibular	N/A	No	22 months	Marginal mandibulectomy	Standard X-ray (panoramic): infiltrative ill-defined osteolysis with destruction of buccal and lingual cortical plates of right posterior mandible; digital volumetric tomography: ill-defined osteolytic changes; CT/MRI: not reported
Satomi et al., 2010 [17]	Case Report	1	14	F	Alveolar	3 × 2 cm	No	10 years	Total excision	Standard X-ray (panoramic): homogeneous soft tissue mass overlying left body of mandible with resorptive defect of alveolar bone and displacement of lower second molar; CT/MRI: not reported
Alkindi et al., 2017 [2]	Case Report	1	27	F	Retromandibular	5 × 4 × 3 cm	N/A	N/A	Excisional biopsy with 5 mm clear margins	Standard X-ray (panoramic): severe bone loss from left mandibular para-symphysis to angle with complete loss of bone surrounding posterior teeth; CT: sub-centimeter axillary and inguinal lymphadenopathy bilaterally, no bony metastatic lesions; MRI: focal solid homogeneous mass with bone destruction extending from canine to retromolar trigone/angle, ramus involvement, lingual and buccal cortical perforation
Tateishi et al., 2016 [18]	Case Report	1	11	F	Alveolar	3 cm	No	18 months	Curettage	Standard X-ray (panoramic): unilocular radiolucent lesion in right anterior mandible with resorption of root of tooth 42 and medial root of tooth 44; CT: well-circumscribed, non-expansile 3 cm osteolytic lesion eroding buccal cortical plate with root resorption; MRI: not reported
Korlepara et al., 2017 [19]	Case Report	1	22	M	Retromandibular	4 × 6 cm	N/A	N/A	Total excision	Standard X-ray (panoramic/OPG): irregular radiolucency extending from distal aspect of tooth 38 to posterior border of ramus, and from sigmoid notch to lower border of mandible; CT (3D): perforation at left ramus region with same extensions as OPG; MRI: not reported
Adachi et al., 2015 [7]	Case Report	1	42	M	Alveolar (mimicking apical periodontitis)	1.8 × 1.1 cm	No	24 months	En bloc resection	Standard X-ray (panoramic): radiolucency at teeth #29 and #30 with unclear lesion boundary; CT: osteolytic lesions with destruction of lingual and buccal cortical plates at teeth #28–30, suggesting aggressive neoplasm; MRI: low intensity on T1-weighted image, enhanced margin on gadolinium-enhanced T1, high signal on STIR
Tripathi et al., 2025 [20]	Case Report	1	28	F	Retromandibular	N/A	No	12 months	Surgical excision (hemimandibulectomy)	Standard X-ray (panoramic): large, multilocular, osteodestructive lesion extending from root of 46 to ramus area with root resorption of 47 and 48; CT: large expansile lesion of right posterior mandible; MRI: not reported
Benites et al., 2025 *(Present case)*	Case Report	1	15	F	Alveolar	3.6 cm	No	12 months	JIAD: total excision and reconstruction	Standard X-ray: poorly defined radiolucent lesion in left anterior-to-premolar mandibular body region.; CT (contrast-enhanced): infiltrative, minimally enhancing, chronically erosive lesion within marrow space of left hemimandible extending from just across midline in mental symphysis to junction of first and second molars, no cervical lymphadenopathy; MRI: incomplete study limited by orthodontic hardware artifact

## 4. Discussion

IMTs are a heterogeneous group of rare neoplasms composed of myofibroblastic and fibroblastic spindle cells accompanied by an inflammatory infiltrate of plasma cells, lymphocytes, and/or eosinophils [1]. The standard treatment strategy for IMTs involves complete surgical resection with negative margins. For unresectable or relapsed cases, systemic therapy is used. Many IMTs exhibit ALK gene dysregulation, which can be targeted therapeutically. Markers, including SMA, vimentin and ALK-1, differentiate IMTs from other spindle cell tumors. In a recent review, crizotinib was found to be the most frequently prescribed agent among the 43 case reports mentioned. In crizotinib-resistant cases, second- and third-generation inhibitors were successful at shrinking these tumors [21]. These ALK inhibitors have shown efficacy for resistant tumors in both adult and pediatric patients [21]. In the future, genomic profiling could help guide personalized treatment plans.

### 4.1. Imaging Characteristics

A critical gap in the current literature on mandibular IMTs is the lack of detailed imaging characterization. Among the 14 cases reviewed, imaging modalities varied considerably: all cases included standard radiographs (panoramic X-rays), but CT was reported in only seven cases, MRI in only three, and no case utilized FDG-PET. The most common radiographic finding was an ill-defined radiolucent or osteolytic lesion, though presentations ranged from well-circumscribed unilocular radiolucencies to multilocular osteodestructive lesions with cortical perforation. This heterogeneity in both imaging utilization and radiographic appearance shows the diagnostic challenge and highlights the need for standardized imaging in the workup of suspected mandibular IMTs. On standard radiographs, mandibular IMTs typically present as ill-defined radiolucent (lytic) lesions, which may mimic periapical pathology, odontogenic cysts, or aggressive malignancies [5,6]. On multidetector computed tomography (MDCT), IMTs typically appear as solid soft-tissue density masses. In a study of 54 IMT patients, all lesions demonstrated soft tissue densities on plain CT, and after contrast administration, all lesions showed persistent enhancement; 72.7% demonstrated heterogeneous enhancement with cystic regions [21]. In the head and neck region specifically, ill-defined margins and calcification were seen more frequently compared to other anatomic sites [21]. CT is particularly valuable for assessing cortical bone destruction, mandibular canal invasion, and the extent of soft tissue involvement, all of which are important for surgical planning. On magnetic resonance imaging (MRI), IMTs typically demonstrate isointense-to-hypointense signals relative to skeletal muscle on T1-weighted sequences and heterogeneously high signals on T2-weighted sequences [21,22]. The fibrous tissue component of the tumor may result in areas of low T2 signals, and delayed enhancement on gadolinium-enhanced sequences have been described, reflecting the fibrotic nature of the lesion [22,23]. MRI is superior to CT for delineating soft tissue extension, perineural spread, and involvement of the inferior alveolar nerve canal [22]. On ultrasound (US), IMTs appear as non-homogeneous solid formations, and US can provide additional characterization regarding whether the lesion is cystic or solid and whether it demonstrates internal vascularity [23,24]. In the series by Masciocchi et al., US detected the presence of a non-homogeneous solid formation in all cases and identified calcifications in a subset. Contrast-enhanced CT and MRI both showed early enhancement associated with multiple peripheral hypertrophic blood vessels, suggesting that IMTs are vascular lesions [24]. Regarding the specific question of whether mandibular IMTs are cystic or solid, the majority of evidence indicates that they are predominantly solid lesions, although heterogeneous enhancement with cystic regions is common. The vascularity of IMTs is variable but generally present, as evidenced by contrast enhancement on both CT and MRI [21,23]. Invasion of the mandibular canal and soft tissue extension should be carefully assessed, as these findings influence surgical planning and may suggest more aggressive biological behavior. Tripathi et al. described an intraosseous IMT of the posterior mandible that demonstrated significant bone destruction on imaging, reinforcing the need for comprehensive cross-sectional imaging in the workup of these lesions [25]. In the present case, contrast-enhanced CT demonstrated an infiltrative, minimally enhancing lesion within the marrow space of the left hemimandible without suspicious peripheral soft tissue enhancement, a pattern consistent with the broader IMT imaging literature [21,22]. The absence of definitive soft tissue invasion beyond the periosteum on CT was an important finding that informed the surgical planning, although MRI was limited by orthodontic hardware artifact.

Regarding metabolic imaging, no mandibular IMT case in the literature has reported 18F-fluorodeoxyglucose positron emission tomography (FDG-PET) findings to date. However, data from IMTs at other anatomic sites indicate that these tumors demonstrate variable but often significant FDG avidity. Dong et al. evaluated FDG-PET/CT findings in a series of IMT patients and reported SUVmax values ranging from 3.3 to 31. Notably, tumors with a predominantly myxoid/vascular histologic pattern tended to show lower uptake, whereas those with compact spindle cell or hypocellular fibrous patterns demonstrated higher metabolic activity [26,27]. In a separate series of hepatic IMTs, Kang et al. found that all lesions demonstrated hypermetabolic activity on FDG-PET/CT. When FDG-PET is performed in the workup of a mandibular lytic lesion, the presence of moderate-to-strong FDG uptake should not be used to exclude IMT from the differential diagnosis, as it does not reliably distinguish IMT from malignant neoplasms. Conversely, FDG-PET may have a role in assessing the extent of disease and monitoring treatment response in cases treated with systemic therapy [28].

### 4.2. Differential Diagnosis

While the radiographic differential diagnosis of a mandibular lytic lesion is broad and includes odontogenic and non-odontogenic pathologies (as discussed in the Introduction), the histopathologic/molecular differential diagnosis of IMT centers on other lesions that share overlapping morphologic features. Differentiating IMTs from other spindle cell tumors and lesions that may mimic them is of paramount importance, as misdiagnosis can lead to inappropriate treatment. The main differential diagnoses for mandibular IMTs include nodular fasciitis, fibrosarcoma (including low-grade fibromyxoid sarcoma), spindle cell/sclerosing rhabdomyosarcoma, aggressive fibromatosis (desmoid tumor), IgG4-related disease, and sarcoidosis. Less common mimics span reactive lesions and other intermediate-grade fibroblastic tumors, such as solitary fibrous tumor and malignant spindle cell neoplasms, including leiomyosarcoma, synovial sarcoma and GIST, each separable from IMT by a targeted immunohistochemical and molecular panel. What unifies the workup across all of these is that ALK, ROS1 and NTRK status, rather than morphology alone, distinguishes IMT from its closest mimics.

In the present case, the histopathologic differential was narrowed by the immunohistochemical profile: ALK (5A4) positivity in 74% of nuclei and SMA positivity, with negativity for AE1/AE3, desmin, myogenin, and CD34. This panel effectively excluded spindle cell/sclerosing rhabdomyosarcoma, aggressive fibromatosis, and fibrosarcoma. The strong ALK positivity also argued against IgG4-related disease and sarcoidosis, both of which are ALK-negative. This therefore illustrates the diagnostic value of an immunohistochemical panel in arriving at a diagnosis of mandibular IMT.

Nodular fasciitis is a benign, self-limiting myofibroblastic proliferation that can closely resemble IMT histologically. Both lesions are composed of spindle cells in a variably myxoid stroma. The key molecular distinction is that nodular fasciitis harbors USP6 gene rearrangements, which are absent in IMTs [29,30]. Coleman et al. demonstrated the diagnostic utility of USP6 rearrangement testing in differentiating nodular fasciitis from IMT, emphasizing that this molecular marker is highly specific for nodular fasciitis and should be employed when diagnosis is uncertain [31]. ALK immunohistochemistry is positive in approximately 50–70% of IMTs but is consistently negative in nodular fasciitis [32,33].

Fibrosarcoma, particularly low-grade fibromyxoid sarcoma and adult-type fibrosarcoma, must be excluded in any spindle cell lesion of the mandible. Fibrosarcomas typically demonstrate greater cytologic atypia, a higher mitotic rate, and a herringbone growth pattern. Molecularly, low-grade fibromyxoid sarcomas harbor FUS::CREB3L2 or FUS::CREB3L1 fusions, which are distinct from the ALK, ROS1, and NTRK fusions found in IMTs [34]. Immunohistochemically, fibrosarcomas lack ALK expression and SMA positivity, which are characteristic of IMTs.

Spindle cell/sclerosing rhabdomyosarcoma is an important and potentially lethal mimic of IMT, particularly in the mandible. Lee et al. reported a case of oral spindle cell/sclerosing rhabdomyosarcoma of the mandible with ALK expression that mimicked an IMT, highlighting the diagnostic pitfall of relying solely on ALK immunohistochemistry [35]. Intraosseous spindle cell rhabdomyosarcomas, particularly those harboring TFCP2 fusions (FUS::TFCP2 or EWSR1::TFCP2), have a predilection for craniofacial bones and are highly aggressive [36,37]. These tumors express desmin, MyoD1, and myogenin, which are markers that are negative in IMTs, and this immunohistochemical panel is essential for distinguishing these entities. The co-expression of ALK and epithelial markers in TFCP2-rearranged rhabdomyosarcomas can further confound the diagnosis if a limited immunohistochemical panel is used [36,38].

Aggressive fibromatosis (desmoid tumor) of the mandible is another rare differential consideration. Seper et al. described aggressive fibromatosis involving the mandible, noting its locally infiltrative behavior and high recurrence rate [39]. Desmoid tumors are driven by CTNNB1 mutations or APC alterations and demonstrate nuclear β-catenin expression, which is absent in IMTs [40]. Unlike IMTs, desmoid tumors lack ALK expression and inflammatory infiltrates.

IgG4-related disease (IgG4-RD) represents a critical differential diagnosis that has gained increasing recognition. IgG4-RD is a systemic fibroinflammatory condition characterized by tumefactive lesions with dense lymphoplasmacytic infiltrates, fibrosis, and obliterative phlebitis [41,42]. The morphologic overlap between IMT and IgG4-RD is substantial, and a subset of IMTs can exhibit elevated IgG4-positive plasma cells and increased IgG4/IgG ratios, further complicating the distinction. Saab et al. analyzed 36 IMTs and found that 15 cases showed IgG4/IgG ratios ≥ 0.10, a cutoff described as supportive of IgG4-RD, demonstrating that the ratio alone cannot reliably discriminate between these two [43]. Taylor et al. identified seven IMTs with morphologic and immunohistochemical features of IgG4-RD, with three patients originally misdiagnosed as IgG4-RD; all were subsequently found to harbor IMT-related gene fusions on next-generation sequencing [41]. The key distinguishing features are: (1) ALK and ROS1 immunohistochemistries, which are positive in IMTs but negative in IgG4-RD; (2) obliterative phlebitis, which is characteristic of IgG4-RD but rare in IMTs; (3) significantly higher IgG4-positive plasma cell counts and IgG4/IgG ratios in IgG4-RD compared to IMTs; and (4) molecular testing for IMT-associated gene fusions (ALK, ROS1, NTRK, RET), which is the definitive discriminator [41,42,44]. Li et al. reported an ALK-1-positive IMT of the thyroid complicated by Hashimoto’s thyroiditis, illustrating the complex interplay between autoimmune inflammation and IMT and the importance of molecular confirmation [45].

Sarcoidosis of the mandible is a rare but important differential diagnosis. Intraosseous sarcoidosis can present as lytic bone lesions of the jaw that mimic neoplastic processes. Cheema et al. reviewed 27 cases of intraosseous sarcoidosis of the jaw and found that these lesions most commonly presented with localized mandibular involvement, with patients exhibiting loose teeth, bone loss, and nonhealing bony defects [46]. Histologically, sarcoidosis is characterized by well-formed, non-caseating granulomas composed of epithelioid histiocytes and multinucleated giant cells, which are morphologically distinct from the myofibroblastic spindle cell proliferation of IMTs [47]. However, the clinical and radiographic overlap can be significant, and biopsy with histopathological examination is essential for differentiation. Sarcoidosis should be particularly considered in patients with systemic features such as bilateral hilar lymphadenopathy, skin lesions, or elevated serum ACE levels [46,48].

### 4.3. Diagnostic Approach: Biopsy and Molecular Confirmation

Given the broad differential diagnosis and significant therapeutic implications of an accurate diagnosis, the approach to tissue sampling in suspected mandibular IMTs warrants careful consideration. Fine-needle aspiration cytology (FNAC), while minimally invasive, has significant limitations in the diagnosis of spindle cell lesions. The cytologic features of IMT on FNAC, including bland spindle cells with inflammatory cells, are nonspecific [49,50]. Stoll and Li reviewed 20 IMT cases with cytology material and noted that while the majority showed bland spindle cells with plasma cells and lymphocytes, focal cytologic atypia was present in seven cases, and the cytologic diagnosis of IMT remained challenging [49]. FNAC alone is generally insufficient for definitive diagnosis because it does not provide the tissue architecture needed for immunohistochemical studies. Core needle biopsy (CNB) is preferred over FNAC for the primary diagnosis of soft tissue tumors, as it provides tissue suitable for immunohistochemistry and molecular testing [34,51,52]. The NCCN guidelines recommend image-guided core needle biopsy as the preferred approach for soft tissue tumors, with incisional (open) biopsy reserved for cases where core needle biopsy yields nondiagnostic findings [34]. For intraosseous mandibular lesions, percutaneous core biopsy under CT guidance or open incisional biopsy may be necessary to obtain adequate tissue. Open biopsy provides the highest diagnostic accuracy (89%) compared to FNAC (82%) and core biopsy (78%) and should be considered when initial biopsy results are inconclusive [53]. The final diagnosis of IMT rests on the integration of immunohistochemistry and molecular testing. The immunohistochemical panel should include ALK, SMA, desmin, myogenin, MyoD1, AE1/AE3, CD34, and β-catenin to systematically exclude the key differential diagnoses [32,33]. ALK positivity is found in approximately 50–70% of IMTs and is the most useful single marker, but ALK-negative IMTs require further molecular workup [32,34]. For ALK-negative cases, testing for ROS1, NTRK, and RET fusions using FISH or next-generation sequencing (NGS)-based RNA fusion assays is recommended [31,34,41]. When IgG4-RD is in the differential, IgG4 and IgG immunohistochemistry with IgG4/IgG ratios should be performed [41,42,43]. USP6 FISH should be considered when nodular fasciitis cannot be excluded morphologically [29,30].

### 4.4. Biological Behavior and Surgical Margins

A central question in the management of mandibular IMTs is whether these tumors behave aggressively and whether wide surgical margins are required. IMTs are classified by the WHO as intermediate-grade neoplasms (rarely metastasizing) within the fibroblastic and myofibroblastic tumor category [34]. While the majority of IMTs behave in an indolent fashion, a subset demonstrates locally aggressive behavior, and rare cases of distant metastasis have been reported [5,54].

In a systematic review of 673 pediatric IMT patients, Raitio and Losty found that recurrence occurred in 20% of cases, and positive tumor margins were a significant risk factor for both recurrence (*p* < 0.0001) and mortality (*p* < 0.0001) [54]. Complete surgical resection (R0) was identified as the strongest predictor of favorable outcomes. However, a multi-institutional study by Rich et al. of 182 IMT patients reported that gross or microscopic positive margins were not associated with increased recurrence, suggesting that aggressive attempts at resection that would compromise form or function may not always be warranted [55,56].

In terms of the head and neck specifically, Ong et al. evaluated 28 patients with head and neck IMTs and found that surgical margins were the primary and independent determinant of survival, followed by tumor size, pseudocapsule status, intralesional necrosis, and Ki-67/ALK overexpression. They recommended radical resection with negative margins as the mainstay of treatment, with post-operative radiotherapy considered for high- and moderate-risk groups [57].

Based on the available evidence, complete surgical resection with negative margins remains the standard of care for mandibular IMTs. However, the decision regarding margin width should be individualized, balancing oncologic adequacy against functional preservation, particularly in anatomically critical regions. The use of the JIAD technique in the present case demonstrates that immediate reconstruction can be successfully integrated with oncologic resection, providing both disease control and functional rehabilitation.

The present patient, a 15-year-old female, is among the youngest in this mandibular IMT series, with only the cases reported by Tateishi et al. and Satomi et al. being younger. Despite the higher recurrence risk reported in pediatric IMT populations (approximately 20%), the present patient achieved disease-free status at 12 months following segmental resection with negative margins and JIAD reconstruction. The rapid functional recovery, transitioning from tube feeds to a full soft oral diet within 7 days and achieving full oral function, compares favorably to the outcomes reported in the reviewed literature, where functional rehabilitation is not consistently described or addressed.

IMTs exhibit one of three histological patterns: myxoid/vascular, compact spindle cell or hypocellular fibrous. The variety in histological patterns is partly responsible for the need for immunohistochemical examination in diagnosing these tumors and to prevent misinterpretation, particularly in the head and neck region. This case offers a look into some of the characteristics and surgical approaches to resecting an IMT, particularly in anatomically problematic regions like the head and neck.

The outcomes of this case are consistent with those reported in the literature. Similar to the majority of reviewed cases, complete surgical excision with clear margins resulted in disease-free survival at follow-up, with no evidence of recurrence at 12 months. What distinguishes this case is the use of the JIAD technique, which allowed for immediate functional and esthetic rehabilitation. While prior cases mostly achieved disease control with excision alone, long-term oral function was not consistently addressed. In contrast, the present patient regained full oral intake within one week and continues to maintain satisfactory oral function with dental rehabilitation.

Some of the literature suggests that not all mandibular IMTs need a segmental resection. Several authors [11,12,13,18] achieved at least short-term disease control with curettage or enucleation at 1–2.5 years of follow-up. These cases predominantly involved well-circumscribed, non-expansile lesions without cortical perforation or soft tissue extension. The present case, by contrast, involved an IMT with transcortical erosion into the soft tissue adjacent to the mandible. This lesion was infiltrative into the medullary space and had thinned out the mandible considerably. Curettage or enucleation would have left not only disease behind but also a very thin mandible prone to fracture in a dentulous young person. The recurrence rate in pediatric IMTs with conservative surgery is 20%, making anything short of a segmental resection inadequate [54]. Curettage or enucleation should be reserved for select IMTs that are well-defined, non-infiltrative lesions without cortical erosion.

## 5. Conclusions

Mandibular IMTs are rare neoplasms that pose significant diagnostic and therapeutic challenges. Clinicians should consider IMT in the differential diagnosis of any large, aggressive, or atypical lytic bone lesion of the mandible, particularly when conventional diagnoses fail to explain the clinical picture. Dedicated imaging with MDCT and MRI is essential for characterizing the lesion and guiding surgical planning. The imaging appearance of a solid, enhancing, ill-defined intraosseous mass should prompt biopsy. Tissue diagnosis should be pursued via core needle biopsy or open incisional biopsy rather than FNAC alone, as adequate tissue is required to distinguish IMT from its mimics. The immunohistochemical panel should include ALK, SMA, desmin, myogenin, MyoD1, and β-catenin at minimum, with IgG4/IgG quantification and molecular testing (FISH or NGS for ALK, ROS1, NTRK, USP6 fusions) as needed. The final diagnosis of IMT requires integration of histomorphology, immunohistochemistry, and molecular biology, as no single marker is pathognomonic.

Several practical recommendations emerge from this review. Complete surgical resection with negative margins remains the treatment of choice, though margin width should be individualized based on tumor biology and anatomic considerations. When feasible, JIAD reconstruction can be an option and may be comparable to conventional excision. JIAD offers the added benefit of the early restoration of oral function, improving patient quality of life, as demonstrated in the present case. Prolonged follow-up is recommended given the potential for local recurrence, which occurs in up to 20% of cases. For unresectable or recurrent tumors, ALK-targeted therapy with agents such as crizotinib represents a promising systemic option. Future studies incorporating genomic profiling may further improve risk stratification and guide treatment strategies.

## Data Availability

The original contributions presented in this study are included in the article. Further inquiries can be directed to the corresponding author.

## Abbreviations

The following abbreviations are used in this manuscript:

IMT	Inflammatory Myofibroblastic Tumor
JIAD	Jaw in a Day
ALK	Anaplastic Lymphoma Kinase
SMA	Smooth Muscle Actin
CT	Computed Tomography
MDCT	Multidetector Computed Tomography
MRI	Magnetic Resonance Imaging
US	Ultrasound
CBCT	Cone Beam Computed Tomography
FDG-PET	Fluorodeoxyglucose Positron Emission Tomography
FNAC	Fine-Needle Aspiration Cytology
CNB	Core Needle Biopsy
FISH	Fluorescence In Situ Hybridization
NGS	Next-Generation Sequencing
IgG4-RD	Immunoglobulin G4-Related Disease
